# Parasite co-invasion by northern pike (*Esox lucius*): molecular evidence and establishment of alien parasites in the Zrebar Lake

**DOI:** 10.1016/j.ijppaw.2026.101262

**Published:** 2026-07-17

**Authors:** Loghman Maleki, Eghbal Gholami, Edris Ghaderi

**Affiliations:** a Department of Biological Sciences, Faculty of Science, University of Kurdistan, Sanandaj, Iran; b Department of Fisheries, Faculty of Natural Resources, University of Kurdistan, Sanandaj, Iran

**Keywords:** *Esox lucius*, *Raphidascaris acus*, ITS, Invasive species, Co-invasive, *Zrebar Lake*

## Abstract

Biological invasions by apex predators can restructure the destination range through ecological turbulence. This study investigates the co-i,markedntroduction and establishment of parasites associated with the invasive northern pike, *Esox lucius,* in Zrebar Lake (Tigris River basin, Iran). A total of 100 specimens of pike were collected during 2020 and 2025, and two alien parasites were found: the monogenean *Tetraonchus monenteron* and the nematode *Raphidascaris acus*. The obtained ITS sequences showed 100% identity with *R. acus* sequences from Anzali (KM047505) and Turkey (KT633862). Compared with populations from the native range, the invasive pike population showed reduced parasite richness, prevalence, and infection intensity. However, these reductions alone are insufficient to explain the invasion success of *E*. *lucius* under the framework of the Enemy Release Hypothesis (ERH), and further evidence is required to evaluate this mechanism. Larval stages of *R. acus* were detected in the non-native cyprinid *Carassius gibelio*, indicating successful completion of the parasite life cycle in the invaded ecosystem. The present study provides evidence for parasite co-invasion and establishment associated with northern pike in Zrebar Lake. It highlights the ecological importance of invasive host–parasite interactions in freshwater ecosystems.

## Highlights

• Co-introduction of two alien helminths with invasive northern pike in Zrebar Lake.
• Molecular phylogeny traced the origin of alien parasites to the Caspian region.
• Alien nematode population was linked to a Caspian–Anatolian lineage of *Raphidascaris acus.*
• Reduced parasite richness and infection levels were observed in invasive pike within the invaded environment.

## Graphical abstract

**Figure:**
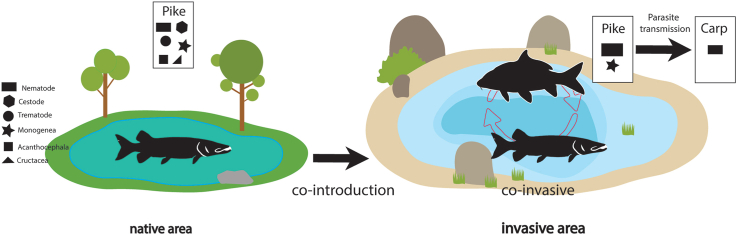

## 1 Introduction

Invasive species are widely recognized as one of the primary drivers of global biodiversity loss across diverse ecosystems. Following their establishment in novel habitats, these species may outcompete or prey upon native flora and fauna, ultimately displacing indigenous organisms through mechanisms such as pathogen transmission, resource competition, and ecological dominance (Simberloff, 2015; Lymbery et al., 2014). The impacts are particularly pronounced when invasive species act as apex predators, thereby disrupting ecological stability through alterations in food-web structure, predation across multiple trophic levels, and the decline or local extinction of native keystone species (Wainright et al., 2021).

One of the main concepts in invasion biology is the Enemy Release Hypothesis (ERH), proposing introduced species may gain a competitive advantage in the novel environments by escaping of their native parasite and pathogen fauna (Torchin et al., 2003; Mitchell and Power, 2003). Reduced parasite richness and infection pressure may increase host fitness, thereby facilitating the establishment and expansion of invasive populations. However, some parasites may be transported together with their introduced hosts, resulting in parasite co-introduction and, in some cases, successful co-establishment in the invaded ecosystem (Lymbery et al., 2014). Alien parasites may subsequently infect native hosts through spillover processes or utilize native species as intermediate hosts, potentially generating additional ecological impacts beyond those caused directly by the invasive host itself (Thieltges and Goedknegt, 2023).

The northern pike (*Esox lucius* Linnaeus, 1758) is a Holarctic species widely recognized as one of the successful invasive fish species worldwide (Dunker et al., 2018). Owing to its high ecological adaptability and apex predatory behavior, this species exerts substantial negative impacts on native fish assemblages and aquaculture systems. In non-native environments, the ecological risks associated with its introduction often outweigh any potential benefits to fisheries, resulting in considerable ecological and economic costs (Cucherousset and Olden, 2011; Muñoz-Mas et al., 2016).

In Iran, the northern pike is native to the coastal waters of the Caspian Sea basin (Coad, 2016). However, it has also been introduced into several non-native regions. The species was first reported from Zrebar Lake in 2016 (Coad, 2016), where it has since established a self-sustaining invasive population that has become the basis of both commercial and recreational fisheries. Since its introduction into Zrebar Lake, the northern pike has adversely affected not only native fish communities but also other vertebrate taxa, including amphibians (Molodi et al., 2024).

Zrebar is a shallow freshwater Lake located within the Tigris River basin in western Iran and is recognized as an internationally important wetland due to its high biodiversity and ecological value (Reyahi-Khoram and Hoshmand, 2012). The lake historically supported a diverse native fish fauna, including species of *Capoeta* and spiny eel, *Mastacembelus mastacembelus* (Banks & Solander, 1794); however, extensive aquaculture activities and intentional fish introductions have substantially altered the composition of the fish community. Currently, several non-native cyprinid fishes, particularly *Alburnus hohenackeri*, *Carassius gibelio*, *Cyprinus carpio*, *Hypophthalmichthys molitrix*, *Ctenopharyngodon idella*, *Hemiculter leucisculus,* and one poeciliid species, *Gambusia holbrooki,* are the main residents of the lake (Esmaeili et al., 2011; Gholami and Shapoori, 2017), whereas populations of native fish species appear to have been displaced. The establishment of the northern pike represents an added ecological pressure for both native communities and parasite transmission dynamics.

The present study aimed to: (i) investigate the helminth parasites co-introduced with *E*. *lucius* into Zrebar Lake, (ii) compare the parasite fauna and infection parameters with those reported from the host native range, (iii) to use molecular techniques to confirm parasite identity, where necessary, and to investigate phylogenetic affinities with geographically related populations based on available ITS rDNA data, and (iv) assess the potential involvement of introduced cyprinid fishes as intermediate hosts in the invaded ecosystem.

## 2 Material and methods

### 2.1 Sampling

A total of 100 northern pike (*Esox lucius*) specimens (40 males, 60 females) were collected by local fishermen from Zrebar Lake in western Iran across two periods. The first sampling was conducted in September 2020, yielded 80 specimens. The second sampling was carried out in October 2025 and included 20 specimens; this reduced sample size was due to limited fish availability. Furthermore, the primary objective of the second sampling was to confirm the persistence of introduced parasites and evaluate potential spillover into sympatric fish hosts rather than establishing a temporal comparison. The examined fish ranged from 32 to 60 (42 ± 5) cm in total length and from 238 to 1657 (637 ± 250) g in body weight.

To evaluate parasite occurrence in sympatric non-native cyprinid fishes co-occurring with northern pike, four cyprinid species were sampled in October 2025. These species have been established in the lake for over three decades. Despite sampling efforts, no native fish were captured. The sampled species included *A. hohenackeri* (n = 7), *C. gibelio* (n = 14), *C. carpio* (n = 6), and *H. molitrix* (n = 3). Because of the limited sample sizes, these data were used only for a qualitative assessment of parasite occurrence and were not used to estimate prevalence or other epidemiological parameters.

All fish were dissected, and their organs were examined for helminth parasites under a stereomicroscope. For morphological analyses, representative nematode morphotypes were preserved in 70% ethanol, while specimens intended for molecular evaluation were fixed in 99% ethanol. Nematodes and monogenean specimens were cleared in temporary lactophenol mounts for morphological study. Identification was performed following Moravec (1994) for adult and larval nematodes, and Galli et al. (2010) for monogeneans. Terminology related to host-parasite invasion concepts follows Blackburn et al. (2011).

### 2.2 Statistical analysis

Prevalence was calculated as the proportion of infected fish among the total number of fish examined. Exact 95% confidence intervals (Clopper–Pearson method) were calculated for prevalence estimates for both the present study and previous studies (Khara et al., 2004, 2007; Mirhashemi et al., 2018), where sample sizes and numbers of infected fish were available. Differences in prevalence among study sites were evaluated using Chi-square tests of independence based on 2 × 2 contingency tables. For pairwise comparisons between Zrebar Lake and other studied lakes (Amir Kelaieh, Chamkhaleh, and Anzali), separate contingency tables were analyzed. Odds ratios (OR) with 95% confidence intervals (CI) were additionally calculated to quantify the magnitude of differences in infection risk between sites.

Bootstrapped 95% confidence intervals (BCa method; 10,000 resamples) for mean intensity were calculated only for the Zrebar Lake population. Similar analyses could not be performed for previously published studies because the original individual parasite count data were not available. Therefore, comparisons of mean intensity among studies were restricted to descriptive interpretation.

Because only a single invasive population was examined, the comparisons with native populations were considered exploratory and interpreted with caution in the context of the Enemy Release Hypothesis (ERH), as variation among invasive populations could not be assessed. Statistical significance was set at P < 0.05. All analyses were performed using R version 4.4.1 (R Core Team, 2024).

### 2.3 Molecular studies

Genomic DNA was extracted from two representatives of adult nematodes infecting *E. lucius*, using a Tissue DNA Extraction Mini Kit (Favorgen, Taiwan) following the manufacturer's instructions. Larval specimens obtained from *C*. *gibelio* were not sequenced due to insufficient material. The sequences obtained in this study have been deposited in GenBank under accession numbers PZ675883 and PZ675884. The ITS1–5.8S–ITS2 region of rDNA was amplified using primers NC5 and NC2 (Zhu et al., 1998). PCR reactions were performed in 20 μL volumes under standard conditions with an initial denaturation at 95 °C for 15 min, followed by 30 cycles of 94 °C for 1 min, 55 °C for 1 min, and 72 °C for 1 min, with a final extension at 72 °C for 5 min. Because the molecular component of the study was intended primarily for preliminary species confirmation and comparison with available regional lineages, sequencing was conducted on representative adult specimens only.

PCR products were purified and sequenced bidirectionally by Microsynth (Switzerland). Obtained sequences were aligned with available representatives of Raphidascarididae retrieved from GenBank using ClustalW in MEGA X. Phylogenetic relationships were reconstructed using Neighbor-Joining (NJ) and Bayesian Inference (BI) approaches. Genetic distances were estimated under the Kimura 2-parameter model in MEGA X.

## 3 Results

A parasitological survey of northern pike revealed two helminth species: a monogenean inhabiting the gill filaments and a nematode recovered from the intestine (Appendix 1). Preliminary morphological examination identified the monogenean as *Tetraonchus monenteron* (Wagener, 1857; Diesing, 1858. Because this parasite is a well-characterized and commonly reported species, its identification was straightforward.

To investigate potential intermediate hosts, four sympatric cyprinid fish species were examined. Larval stages of *R*. *acus* were recovered from the body cavity of *C*. *gibelio*, whereas no larvae were detected in the other examined cyprinid species. This finding indicates that *C. gibelio* serves as an intermediate host for *R. acus* in Zrebar Lake.

### 3.1 Molecular study

**Fig. 1:**
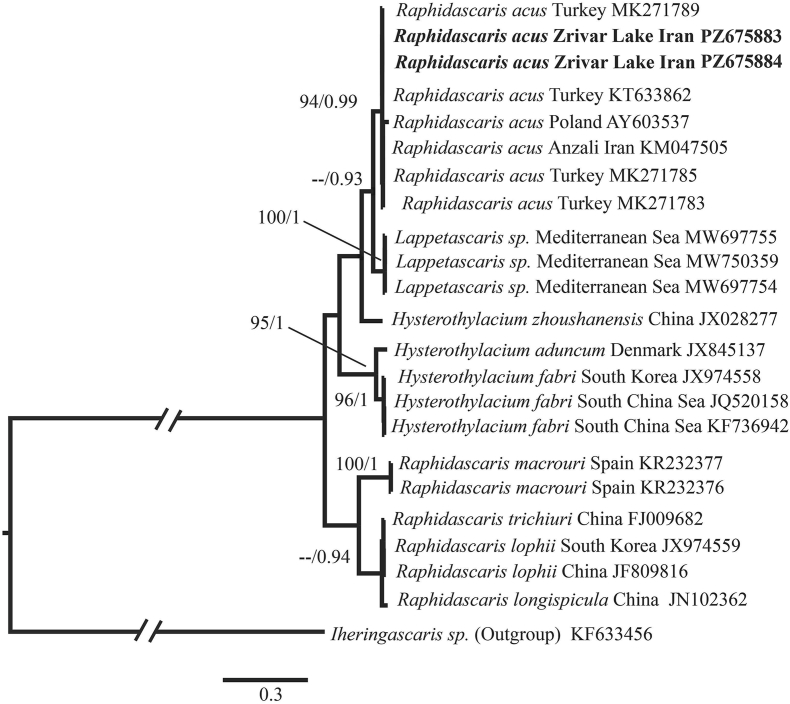
Phylogenetic tree resulting from sequence analysis of the ITS1 region, 5.8S gene, and ITS2 of ribosomal DNA from nematodes of the family Raphidascarididae, based on Neighbor-Joining (NJ) and Bayesian Inference (BI) methods. Numbers on the branches represent bootstrap values from the NJ method and posterior probabilities from the Bayesian analysis, respectively. Specimens obtained from the present study are indicated in bold, and *Iheringascaris* sp. was used as the outgroup.

3.1. Morphological examination initially allowed the nematode specimens to be assigned only to the genus *Raphidascaris*. Therefore, ITS rDNA sequencing was performed to confirm the species identity of the collected specimens. A 936-bp ITS rDNA sequence was generated and deposited in GenBank under accession numbers PZ675883 and PZ675884. BLAST analysis revealed 100% sequence identity with *R. acus* sequences from Anzali (KM047505) and Turkey (KT633862). Phylogenetic analyses using both Neighbor-Joining and Bayesian Inference placed the Zrebar specimens within the well-supported *Raphidascaris acus* clade together with available reference sequences from Iran, Turkey, and Poland (Fig. 1). Pairwise K2P genetic distances among *R. acus* sequences were low (0–0.003), whereas substantially greater divergence was observed between *R. acus* and other available species of *Raphidascaris*. These results provide molecular support for the identification of the examined specimens as *R. acus*.

### 3.2 Ecological study

The overall prevalence of *R. acus* infection in *E. lucius* was 20% (20/100; 95% CI: 13.2–28.8%). However, a significant temporal increase was observed, from 17.5% (14/80; 95% CI: 10.0–27.1%) in 2020 to 40% (8/20; 95% CI: 19.1–63.9%) in 2025 (χ^2^ = 5.3, df = 1, p = 0.02). In pairwise lake comparisons, prevalence in Zrebar Lake did not differ significantly from that in Amir Kelaieh (χ^2^, p > 0.05; OR = 0.68, 95% CI: 0.35–1.31), indicating comparable infection risk. In contrast, Zrebar showed significantly lower prevalence than Chamkhaleh Lake (χ^2^, p = 0.004), with odds of infection nearly 2.4-fold lower (OR = 0.42, 95% CI: 0.22–0.79). The most marked difference was between Zrebar and Anzali, where Zrebar had a significantly lower prevalence (χ^2^, p < 0.001) and approximately 2.9-fold reduced odds of infection (OR = 0.35, 95% CI: 0.19–0.64).

For the alien monogenean *T***.** *monenteron*, the overall prevalence in *E. lucius* was 30% (6/20; 95% CI: 11.9–54.3%). Notably, monogenean infections were recorded only during the second sampling campaign, as the first survey focused exclusively on nematodes. In pairwise prevalence comparisons, infection odds in Zrebar Lake were higher than in Amirkelaye (OR = 1.66, 95% CI: 0.53–5.19) and Chamkhaleh (OR = 2.21, 95% CI: 0.77–6.34), but neither difference reached statistical significance (χ^2^ = 0.82, P = 0.36 and χ^2^ = 2.34, P = 0.13, respectively). In contrast, infection odds in Zrebar were significantly lower than in Anzali Lagoon (OR = 0.28, 95% CI: 0.10–0.78; χ^2^ = 6.08, P = 0.014). **S**tatistical comparisons of mean intensity across study sites were not feasible, as the original individual parasite counts—necessary for estimating variance and computing bootstrapped confidence intervals—were unavailable for previously published datasets. Therefore, intensity comparisons were restricted to descriptive interpretation. Despite this limitation, the mean intensity of *T. monenteron* in Zrebar Lake appeared lower than that reported from the other studied localities.

## 4 Discussion

The northern pike, *E*. *lucius*, is recognized as a highly parasitized fish species throughout its native distribution range. Previous studies have reported 18 parasite species in Canada (Watson and Dick, 1980), 42 helminth species in Poland (Morozińska-Gogol, 2019), 23 species in Russia (Liberman et al., 2019), 10 species in Iran (Khara et al., 2004; Mirhashemi et al., 2018), and 6 species in Turkey (Öztürk et al., 2000). In the southern Caspian Sea basin, which likely represents the source region of the introduced population, more than 10 helminth species have been recorded from *E. lucius* (Table 1). Among all reports, *R*. *acus* and *T*. *monenteron* are considered core parasite species due to their consistent presence, high prevalence and abundance.

**Table 1:** Helminth parasite found in *Esox Lucius* from Iran. Comparison among species richness and prevalence (P) and mean intensity of infection (MI) in invasive and native hosts. n = number of examined fish.

Parasite taxon		This study n = 80 (2020), n = 20 (2025)		Khara et al. (2004) n = 78		Khara et al. (2007) n = 123		Mirhashemi et al. (2018) (n = 128)	
	locality	Zarivar Lake^a^		Amir Kelaieh		Chamkhaleh		Anzali	
	Parasite species	P 95% CI	MI (bootstrap CI 95%)	P 95% CI	MI	P 95% CI	MI	P 95% CI	MI
Acanthocephalan	*Corynosoma stramosum*					0.8 (0.14 – 4.49)	2		
Annelid	*Piscicola* sp.			1.2 (0.22–6.91)	1				
Cestode	*Triaenophorus crassus*			9 (4.17 – 17.78)	2.7	8.9 (5.06 – 15.35)	8.4		
Crustacean	*Argulus* sp.			3.8 (1.33 – 10.68)	1.33				
Crustacean	*Lernaea cyprinacea*			2.6 (0.71 – 8.87)	5	0.8 (0.14 – 4.49)	2	3 (1.21– 7.89)	
Monogenean	*Tetraonchus monenteron*	30 (11.9–54.3%).	5.82 (2.45–11.09	20.5 (12.43– 31.35)	12	16.2 (10.83 – 23.68)	5 ± 4	60 (51.40– 68.32)	40 ± 41
Nematode	*Eustrongylides excisus*			2.6 (0.71 – 8.87)	1			2.3 (0.80– 6.68)	
Nematode	*Raphidascaris acus*	20 (13.2–28.8).	2.13 (1.53–2.93)	26.9 (18.4–37.4)	8.7	37.4 (29.37 – 46.14)	5.2 ± 8.4	41.4 (33.05– 50.27)	
Nematode	*Rhabdochona helichi*							8 (4.29– 13.77)	
Nematode	*Camallanus lacustris*			6.5 (2.76 – 13.94)	1.8	1.6 (0.45 – 5.74)	2 ± 1		
Trematode	*Diplostomum spathaceum*			7.7 (3.61– 15.64)	3.6	3.2 (1.27 – 8.07)	1.2 ± 2	23 (15.99– 30.95)	2.6
Trematode	*Rhipdocotyle illense*							9 (5.43– 15.82)	

^b^ Values are presented as Mean ± SD.

a The Zrebar Lake population represents the introduced (invasive) range of *Esox lucius*, whereas all remaining localities belong to the native Caspian Sea basin distribution range.

In contrast, the invasive population of *E. lucius* in Zrebar Lake exhibited a markedly reduced helminth diversity, with only two parasite species detected. The absence of native parasite species in northern pike also suggests that there is currently no evidence of parasite spillback in the invaded ecosystem (Kelly et al., 2009). Furthermore, both prevalence and infection intensity were generally lower than those reported from native populations. Although these observations are broadly consistent with predictions of the Enemy Release Hypothesis (ERH) (Torchin et al., 2003; Mitchell and Power, 2003), the present study does not directly evaluate whether reduced parasite burden confers a competitive or ecological advantage to the invasive host population. Therefore, the role of ERH in facilitating the successful establishment of northern pike in Zrebar Lake warrants further consideration.

Molecular analyses confirmed that the nematodes recovered from northern pike in Zrebar Lake were *R. acus*; the obtained ITS sequences clustered with previously published *R. acus* sequences. Although the observed phylogenetic pattern is consistent with the taxonomic assignment of the specimens, the ITS marker provides limited resolution for evaluating population-level relationships. This pattern is consistent with the hypothesis that the parasite was introduced, together with its invasive host, from the Caspian Sea. However, the molecular dataset and geographic sampling of the present study do not permit robust conclusions regarding the precise origin of northern pike and associated parasites into Zrebar Lake.

*Raphidascaris acus* shows a relatively high specificity toward its definitive host, the northern pike, *E*. *lucius* (Smith, 1986). In the invaded lake ecosystem, this parasite is considered exotic because no alternative definitive hosts are present other than *E. lucius*, and spillback transmission from native fishes to the introduced pike population appears absent. These observations suggest that the parasite has successfully passed through the stages of co-introduction and co-establishment and has now reached the co-invasive stage (Blackburn et al., 2011; Díaz-Morales et al., 2025).

Although *R. acus* is host-specific to its definitive host, it is more generalist regarding intermediate hosts, utilizing several cyprinid fish species (Moravec, 1994). In the present study, larval stages were recovered from the body cavity of *C. gibelio*, indicating its role as an intermediate host in the invaded ecosystem. Because *C. gibelio* and likely *C. carpio* constitute important prey items for northern pike, their continuous availability in the lake may facilitate completion of the parasite life cycle. Previous studies have reported pathogenic effects of this nematode in intermediate hosts (Moravec, 1994; Koubková et al., 2004), although its impact on local fish populations requires further investigation.

In the present study, the monogenean *T*. *monenteron* was also identified, indicating that it was co-introduced with its invasive host, northern pike, into Lake Zrebar, Iran. Lymbery et al. (2014) identified monogeneans as one of the parasite groups frequently involved in co-introduction events associated with translocated fish hosts. The successful establishment of monogeneans is likely linked to their direct life cycle, which facilitates transmission in newly invaded environments. Among introduced helminths, monogeneans are reported most frequently, followed by nematodes, digeneans, cestodes, and acanthocephalans. Subsequent studies have continued to document additional cases of nematode and co-introduction in aquatic animals (e.g., Lombardo et al., 2022; Rehbein et al., 2024), indicating that parasite co-introduction remains an ongoing component of biological invasion.

Nematodes may benefit from a broader host range and the presence of a resistant external cuticle, which enhances survival under variable environmental conditions. In contrast, cestodes and digeneans are reported less frequently, despite likely being co-introduced with their hosts. Their lower establishment success may be related to their complex life cycles, which require multiple and often specific intermediate hosts in the invaded ecosystem.

## Funding

The project was financially supported by the 10.13039/501100008973University of Kurdistan through research grant no. 1401 was awarded to the first author (L.M.) as the project supervisor.

## CRediT authorship contribution statement

**Loghman Maleki:** Conceptualization, Data curation, Formal analysis, Methodology, Resources, Supervision, Writing – original draft, Writing – review & editing. **Eghbal Gholami:** Formal analysis, Investigation, Methodology, Software, Writing – original draft. **Edris Ghaderi:** Software, Writing – review & editing.

## Declaration of competing interests

The authors declare that they have no conflict of interest.

## Appendix A Supplementary data

Appendix 1 Live adult specimen of *Raphidascaris acus* recovered from the intestine of invasive northern pike (*Esox lucius*) collected from Zrebar Lake, Iran, showing active movement shortly after dissection.
